# Biofabrication of Sodium Alginate Hydrogel Scaffolds for Heart Valve Tissue Engineering

**DOI:** 10.3390/ijms23158567

**Published:** 2022-08-02

**Authors:** Yannick Rioux, Julie Fradette, Yvan Maciel, André Bégin-Drolet, Jean Ruel

**Affiliations:** 1 Department of Mechanical Engineering, Faculty of Science and Engineering, Université Laval, 1065 Avenue de la Médecine, Quebec City, QC G1V 0A6, Canada; yannick.rioux.1@ulaval.ca (Y.R.); yvan.maciel@gmc.ulaval.ca (Y.M.); andre.begin-drolet@gmc.ulaval.ca (A.B.-D.); 2 Centre de Recherche en Organogénèse Expérimentale de l’Université Laval/LOEX, Centre de Recherche du CHU de Québec-Université Laval, 1401, 18e rue, Quebec City, QC G1J 1Z4, Canada; julie.fradette@fmed.ulaval.ca; 3 Department of Surgery, Faculty of Medicine, Université Laval, Quebec City, QC G1S 4L8, Canada

**Keywords:** biofabrication, scaffold, hydrogel, sodium alginate, aortic valve, sacrificial ink, 3D printing, carbohydrate glass

## Abstract

Every year, thousands of aortic valve replacements must take place due to valve diseases. Tissue-engineered heart valves represent promising valve substitutes with remodeling, regeneration, and growth capabilities. However, the accurate reproduction of the complex three-dimensional (3D) anatomy of the aortic valve remains a challenge for current biofabrication methods. We present a novel technique for rapid fabrication of native-like tricuspid aortic valve scaffolds made of an alginate-based hydrogel. Using this technique, a sodium alginate hydrogel formulation is injected into a mold produced using a custom-made sugar glass 3D printer. The mold is then dissolved using a custom-made dissolving module, revealing the aortic valve scaffold. To assess the reproducibility of the technique, three scaffolds were thoroughly compared. CT (computed tomography) scans showed that the scaffolds respect the complex native geometry with minimal variations. The scaffolds were then tested in a cardiac bioreactor specially designed to reproduce physiological flow and pressure (aortic and ventricular) conditions. The flow and pressure profiles were similar to the physiological ones for the three valve scaffolds, with small variabilities. These early results establish the functional repeatability of this new biofabrication method and suggest its application for rapid fabrication of ready-to-use cell-seeded sodium alginate scaffolds for heart valve tissue engineering.

## 1. Introduction

Valvular heart diseases (VHD) are characterized by a loss of valve functionality. Their prevalence has increased over the years due to the ageing of the population [1,2,3,4]. Higher morbidity and mortality are observed for patients with VHD [3,5,6]. The current main treatment is transcatheter aortic valve replacement [7]. Aortic stenosis is the most frequent valvular heart disease and is defined by a narrowing of the passage of blood through the valve during ejection of blood into the aorta (i.e., systolic phase). Calcific aortic valve stenosis, characterized by calcium deposition in the valve leaflets making them stiffer, is the most common type of stenosis [8,9]. Stiffer leaflets generally lead to return of the blood flow into the left ventricle (aortic regurgitation) during the filling of the left ventricle (i.e., diastolic phase). For the same amount of calcification, women tend to have higher aortic valve stenosis severity, which is related to the combination of calcification and valvular fibrosis (valve leaflets thickening) [10]. However, men have a twofold increased risk of developing aortic stenosis [4]. A noteworthy fact is that congenital heart valve diseases affect 1 to 2% of all newborns [11,12]. Most of the cases are related to the presence of a bicuspid aortic valve, characterized by two functional leaflets rather than three, which will eventually lead to the early development of aortic stenosis.

To meet the need for valve replacement, the current solution is to replace the defective valve with a mechanical or biological substitute. However, existing mechanical prostheses lead to an increase in blood coagulation (thrombosis) and rupturing of red blood cells (hemolysis). This is due, respectively, to the presence of foreign surfaces, for which patients need to take lifelong anticoagulation therapy, and to the non-physiological valve geometry that induces high shear stress on blood cells [13,14]. Bioprosthetic valves, homograft valves, or those made using xenogeneic materials provide a better anatomical geometry and lower thrombogenicity [13,15], although chronic inflammation caused by residual immunogenicity/immunogenic materials, the lack of remodeling, and the earlier calcification of the leaflets [9,14,16,17,18] are observed. Thus, the lifespan of these substitutes is limited to between 10 and 20 years due to valve degradation. This durability is insufficient, especially in the case of pediatric patients [19,20,21].

Decellularized xenografts and homografts have made their way into multiple clinical studies as an appealing “off-the-shelf” solution with good valve performance in the short term [14,22]. Decellularized xenografts have shown remodeling and growth potential following implantation, with an observed repopulation by autologous cells. Unfortunately, decellularized xenografts still led to a high risk of immune response, especially in young individuals with a more responsive immune system compared to elders. The risk of potential xenogeneic disease transmission is also still present. These problems eventually lead to valve functionality degeneration in the long term [23,24,25,26] and are likely due to incomplete decellularization. Decellularized homograft valves have been associated with tissue remodelling [27,28] and excellent physiological functionality in the long term [29,30,31,32,33,34]. However, the availability of homograft valves is limited compared to xenograft valves, and potential residual immunogenicity towards decellularized homograft valves also remains [35]. In addition, the decellularization processes affect biomechanical stability in both homograft and xenograft valves [36].

Tissue-engineered heart valves (TEHV) have the potential to provide valve substitutes with remodeling, regeneration, and growth capabilities. In addition to being a replacement substitute, these can also act as models to study diseases and drug treatments to better understand the physiological and pathological mechanisms of the aortic valve. As a response to the various problems related to decellularization, bioresorbable biomaterials and 3D-printed TEHVs have recently been developed [37,38,39,40,41,42,43]. Bioresorbable biomaterials have shown rapid, cost-effective, reproductible, and consistent fabrication of heart valve scaffolds, allowing rapid cellularization, extracellular matrix (ECM) deposition, and scaffold degradation after implantation [37,38,39,40]. Furthermore, new 3D bioprinting techniques have led to the engineering of personalized complex heart valve scaffold geometries containing ECM proteins and living cells [41,42,43]. In both cases, further investigations on the durability of the structural integrity of TEHVs and on the degradation/remodelling of biomaterials, which are often synthetic polymers, will be needed before translating these models towards clinical trials [14,22].

Alginate is a natural biocompatible polymer available at low cost that can be used to make macroporous hydrogels with cell-affinity domains, controllable degradation, and easily tunable mechanical properties [44,45,46,47]. Therefore, it gained interest in recent studies for the fabrication of heart valve scaffolds [41,43,48,49]. These studies primarily focused on the 3D bioprinting of tri-leaflet heart valves featuring great shape fidelity and cell survival.

In this study, we present a new approach for the rapid fabrication of complex heart valve scaffolds for TEHVs made of sodium-alginate hydrogel. The approach is based on a simple low-cost casting method using sugar glass molds. We present data indicating that our developed methodology led to excellent early results in the manufacturing reproducibility of a native-like complex geometry of heart valve scaffolds. Thus, we consider that this new biofabrication method could represent a solution in the manufacturing of TEHVs customized to the patients. In an in vitro approach, autologous cells and ECM proteins could easily be added homogeneously into the hydrogel formulation prior to casting the scaffold, thereby avoiding the immunogenic issues associated with decellularization and the complexity of cellularization.

## 2. Results and Discussion

### 2.1. Fabrication of the Aortic Valve Scaffolds

**Figure 1:**
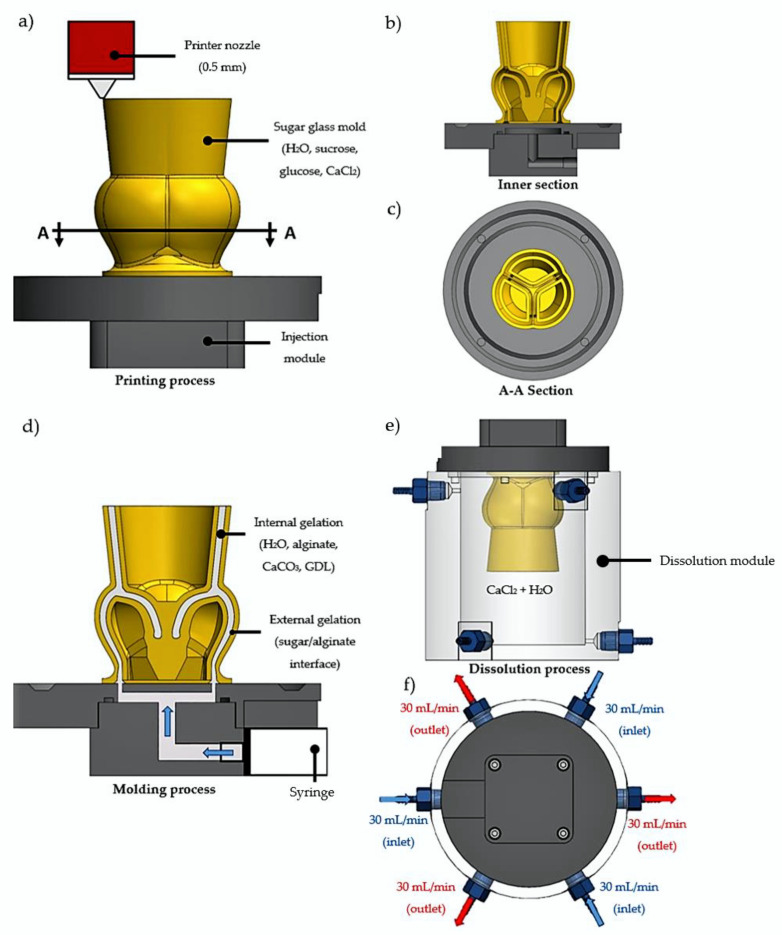
Schematic of the aortic valve scaffold fabrication. (**a**) Aortic valve sugar glass mold loaded with CaCl_2_ and printed with a 0.5 mm custom printer nozzle and a 0.2 mm layer height. (**b**) Inner section of the mold. (**c**) A-A section of the mold showing the interior at the mid-sinus. (**d**) Molding process of the aortic valve scaffold using the syringe loaded with the sodium-alginate formulation containing the sodium alginate loaded with GDL and CaCO_3_ for internal gelation. External gelation occurred at the sugar/alginate interface. (**e**) Transferring the mold injected with the sodium-alginate solution into the dissolution module. (**f**) For the dissolution of the sugar mold, a 90 mL/min total (inlet/outlet) flow of water supplemented with CaCl_2_ was performed.

**Figure 2:**
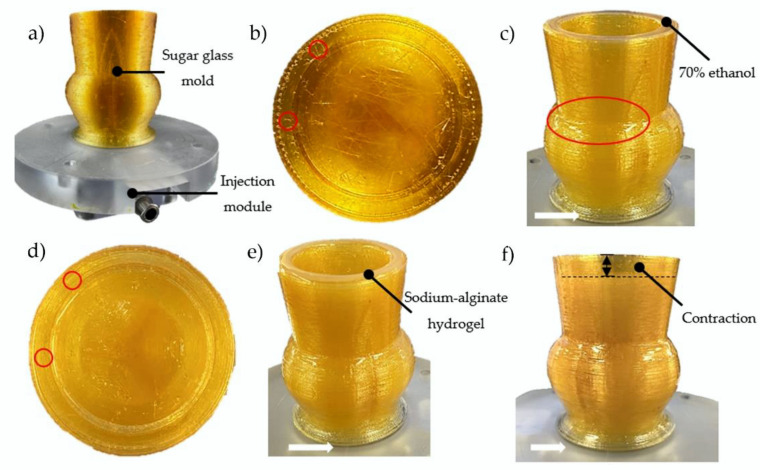
Injection process of the hydrogel scaffold formulation. (**a**) Side view of the mold printed on the injection module. (**b**) Top view of the mold. Small sugar glass filaments were observed after printing (red circles). (**c**) After a short placement in an incubator (37 °C, 100% relative humidity), the surface of the mold dissolved slightly (glossy finish, outlined in red). The mold was then filled with 70% ethanol. (**d**) Few filaments were left after their dissolution with the ethanol solution (red circles). (**e**) Mold filled with the sodium-alginate hydrogel solution. (**f**) The hydrogel scaffold contracted after gelation compared to (**e**). White arrows: no leakage was observed at the base of the mold at any step.

A total of nine sugar glass aortic valve molds composed of sucrose, dextrose, CaCl_2_, and distilled water were successfully printed on their separate injection module (Figure 1a–c and Figure 2a). The total printing time for each mold was 46 h. The relative simplicity and low converting cost of a commercial 3D printer into a sugar glass 3D printer compared to bioprinters allows the use of many printers to accelerate the production rate of molds. Because the molds are made and stored prior to casting with a fresh hydrogel formulation, the printing time of the molds does not influence the scaffold stability. Before casting of the alginate scaffolds, the molds were placed inside a culture incubator (37 °C, 100% relative humidity) for 3 min to slightly dissolve the sugar glass and weld the sugar layers together to improve the sealing of the mold (Figure 2c). This prevented leaking of the hydrogel solution in the next step. Then, prior to casting, the molds were filled with a 30 mL syringe loaded with 70% ethanol solution using the injection module (Figure 2c) for 4 min to remove the unwanted small sugar filaments left by the printer head during the printing process and at the same time to check the sealing of the mold (Figure 2b). Such filaments could possibly trap bubbles or make holes in the scaffolds. After incubation and rinsing, the number of filaments was greatly reduced (Figure 2d). No leakage of the 70% ethanol solution was observed for the nine replicates during the dissolution of the filaments, which indicates that the more viscous hydrogel formulation should not leak during casting (Figure 2c). When cells will be added in the hydrogel formulation optimized for cells in future works, rinsing with ethanol will allow the disinfection of the molds. Prior to alginate casting, a waiting time of 10 min was necessary to let the ethanol evaporate.

A sodium-alginate hydrogel solution supplemented with CaCO_3_ + GDL gelation agents was used for casting of the scaffolds. CaCO_3_ has very low solubility in distilled water, which allows its uniform distribution throughout the scaffold. GDL was used to initiate the gelation by dissociating CaCO_3_ into Ca^2+^ ions, which crosslinked the alginate (internal gelation) and formed a hydrogel. The gelation time was controlled by adjusting the concentrations of CaCO_3_ and GDL and was selected to allow enough time for the hydrogel formulation to be molded before gelation occurred. The molds were filled with the freshly made hydrogel formulation by the bottom, using the injection module (Figure 1d). The low-viscosity alginate used was essential to fill the molds completely and prevent bubbles from being trapped in the hydrogel. Filling the molds from below (Figure 2d) also helped to prevent the trapping of air bubbles during gelation of the hydrogel. Any remaining sugar filaments from the mold were easily dissolved by the highly aqueous hydrogel formulation. The latter would also normally dissolve the highly soluble sugar glass molds. However, a few seconds after the injection, the fast dissolution of the molds at the alginate-sugar interface released calcium ions from the sugar molds, which crosslinked with the sodium alginate (external gelation) to form a membrane at the interface that effectively stopped the dissolution of the molds by the hydrogel formulation (Figure 1d and Figure 2e,f). After 7 min of gelation, there was very little dissolution of the sugar glass molds (Figure 2f). Visually, the scaffold contracted by the amount shown in Figure 2f (dotted line).

**Figure 3:**
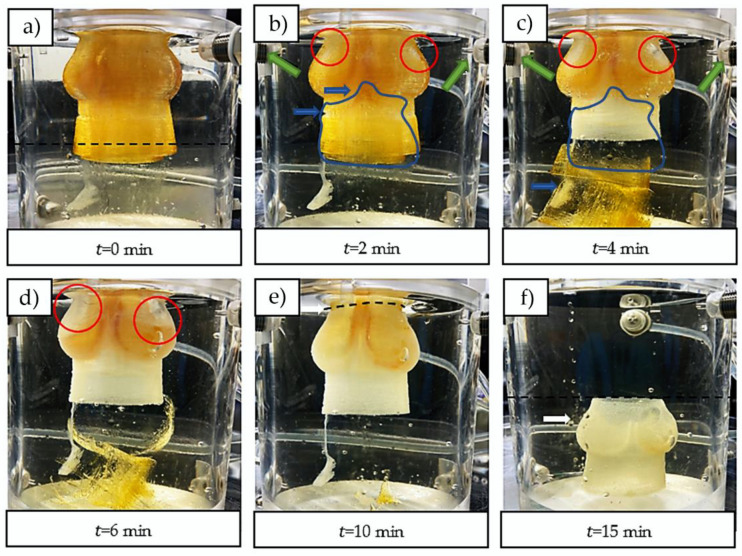
Dissolution process of the sugar glass mold and final gelation step of the scaffold. (**a**) t=0 min, fresh CaCl_2_ solution above the dashed line and dissolved sugar at the bottom (yellowish liquid). (**b**) t=2 min, the inlet jets of fresh CaCl_2_ solution (green arrows) made holes in the sugar glass mold (red circles). The blue arrows and blue outlined area show the dissolving inner structures that are falling to the bottom. (**c**) Dissolution of the mold at t= 4 min. (**d**) Dissolution of the mold at t= 6 min. (**e**) t=10 min, the scaffold was manually cut with a scalpel at the site represented by the dotted line. (**f**) t=15  min, the dissolution and gelation processes were completed.

**Figure 4:**
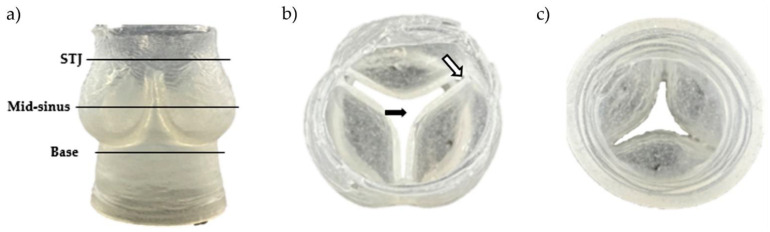
Resulting aortic valve scaffold made of sodium-alginate hydrogel. (**a**) Side view with three regions of interest (STJ: sino tubular junction). (**b**) Top view. The black arrow shows the opened central area, and the white arrow shows the leaflet thickness and the spacing at the commissure. (**c**) Bottom view.

The filled molds were then transferred into a custom-made dissolution module that produces a constant influx of CaCl_2_ solution to complete the external gelation and dissolve the sugar (Figure 1e,f). The constant flux of the dissolution module improved the dissolution rate of the sugar glass mold compared to dissolution in a static aqueous solution by minimizing the sugar concentration inside the dissolution module. Additionally, it allowed the use of a less-concentrated CaCl_2_ solution by ensuring a fresh access to Ca^2+^ ions. The three inlet jets, located near the mid-sinus region (Figure 3b,c), provided a good perfusion of the Ca^2+^ ions into the scaffold. The dissolved sugar was aspirated through the three outlets located at the bottom of the dissolving module. At t=0 min (Figure 3a), the upper section was primarily filled with fresh CaCl_2_ solution, and the dissolved sugar went to the bottom of the module (yellowish lower section), which helped to accelerate the dissolution. At t=2 min (Figure 3b), the three inlet jets located at the top helped to break through the mold, which contributed to ameliorate perfusion of the CaCl_2_ solution inside the mold, and to accelerate the dissolution of the inner structure. At t=6 min (Figure 3d), the external surfaces of the mold were completely dissolved and the perfusion of the CaCl_2_ solution through the scaffold was easily observable. At t=10 min (Figure 3e), the scaffold was manually cut using a scalpel, which facilitated the dissolution of the remaining sugar inner structures of the mold. At t=15 min (Figure 3f), the dissolution was completed, and the aortic valve scaffold fell to the bottom at one point between t=10 and 15 min. A uniform yellowish color throughout the complete dissolution module was observed, corresponding to an estimated concentration of sugar of 2% *w*/*v* (approximately 30 g for the sugar glass mold divided by 1500 mL for the total volume of CaCl_2_ solution in the system). Figure 4 shows one of the resulting aortic valve scaffolds. The total manufacturing time of one aortic valve hydrogel scaffold (dissolution and internal/external gelation, excluding the printing time of the aortic valve mold) was 25 min. In future work, during which cells could be added to the alginate hydrogel, adjusting the inlet/outlet flow rate of the dissolving module to minimize the CaCl_2_ concentration should help to prevent cell death [50,51]. Adjusting the inlet/outlet flow could represent an interesting way to maximize cell access to nutrients from the culture media and the serum during the dissolution process with better perfusion through the scaffolds. Finally, to minimize the sugar concentration effects on cells in future research, the three outlet flows should be evacuated.

### 2.2. Geometric Assessment of the Aortic Valve Scaffolds Indicates Very Good Preliminary Repeatability

**Figure 5:**
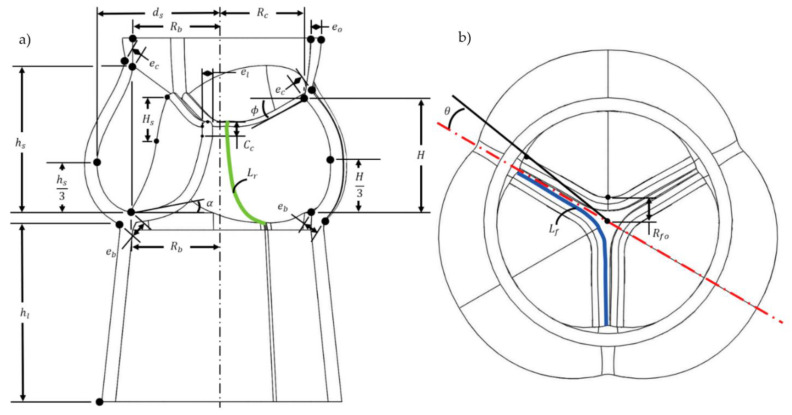
Geometry and parameters of the aortic valve scaffold model. (**a**) Inner section. (**b**) Top view.

**Figure 6:**
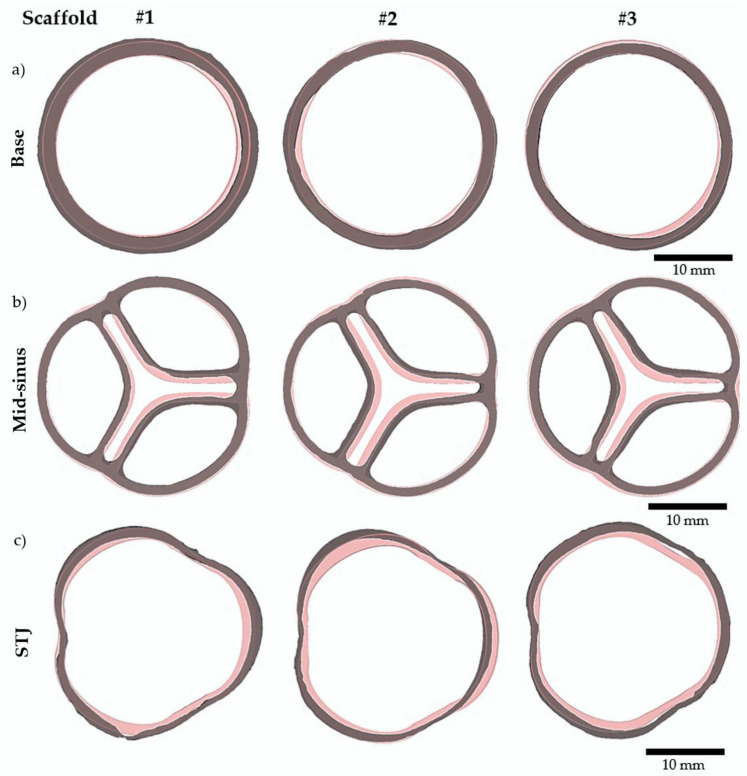
Three aortic valve scaffolds were compared to the original computer-designed model. Images generated after 3D reconstruction of micro-CT scans of *n* = 3 scaffolds are shown in grey. The original computer-designed model to which a uniform contraction of 13% was imposed is shown in pink. (**a**) Base slices. (**b**) Mid-sinus slices. (**c**) STJ (sino tubular junction) slices.

Figure 4 shows one of the resulting aortic valve scaffolds. The center orifice area was larger as well as the spacing at the commissures (Figure 4b) compared to the original computer-designed model (Figure 5b and Figure 6a). This could be caused by a non-linear contraction of the aortic valve scaffolds during the dissolution of the molds where the dissolution rate of sugar is not the same in all parts of the mold (Figure 3d). Therefore, the support of the scaffold was consistently changing at the same time as the scaffold was contracting, leading to the non-linear contraction.

To evaluate the preliminary reproducibility of the present biofabrication method, three aortic valve scaffolds were scanned by micro-CT and reconstructed in 3D. Figure 6 compares the scanned scaffolds to the original computer-designed model with a uniform imposed contraction of 13%, which seemed to best correspond to the contracted scaffolds after the complete gelation (internal and external). Three slices located at the mid-sinus, sino tubular junction (STJ), and the base (Figure 4) served as a reference for comparison (Figure 6). The scaffolds bases matched well the original model base (Figure 6a). At the mid-sinus, the outermost wall of the reconstructed scaffolds matches the one of the original valve (Figure 6b). However, the center orifice was larger. Variations were more important at the sino tubular junction (Figure 6c). Overall, the scaffolds have the same shape as the original model, and the geometric repeatability between scaffolds was very good.

### 2.3. Hydrodynamic Properties of the Aortic Valve Scaffolds

**Figure 7:**
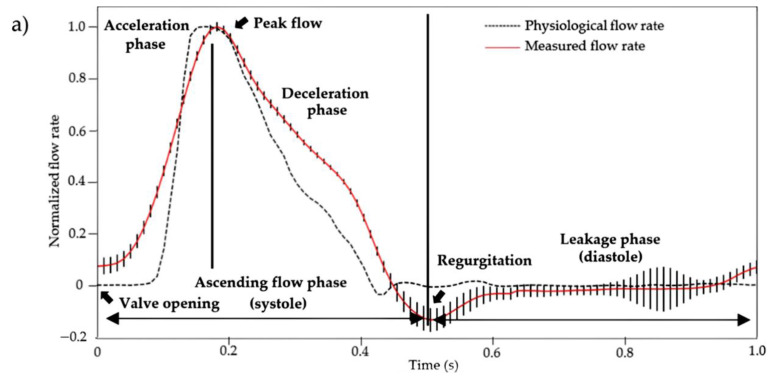

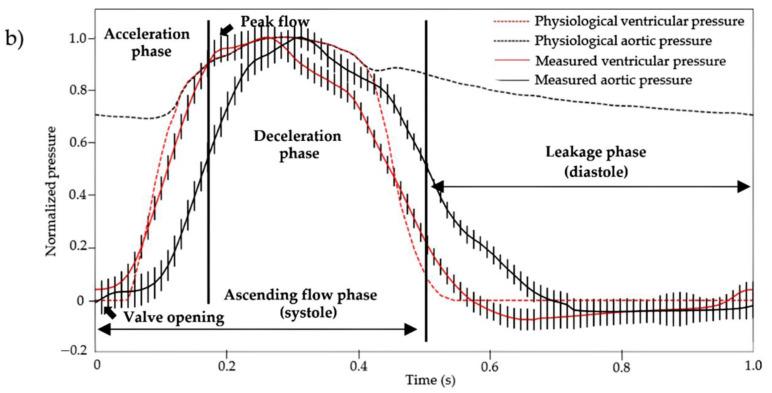
(**a**) Average measured flow rate and (**b**) pressures (aortic and ventricular) normalized profiles (*n* = 12 cardiac cycles) at 60 BPM. These were obtained using a custom-made cardiac bioreactor on three aortic valves scaffolds (measured data curves) and compared to the physiological profiles (physiological value curves) [53]. The vertical bars represent the standard deviation at each acquisition time (100 Hz) of the average profiles of the three aortic valve scaffolds.

Several aortic valve scaffolds were fabricated (not shown) to optimize the concentrations of the gelling agents in the fabrication method to obtain a final scaffold with properties similar to the elastic modulus of native soft cardiac human tissues (~40–110 kPa) [52]. The compression test results (60.84 ± 24.36 kPa), performed on three final aortic valve scaffolds made using the fabrication method presented in this study, showed that the scaffolds had indeed an elastic modulus within the range expected for native soft cardiac human tissues. This was essential to ensure the proper mechanical response of the scaffolds to the cardiac bioreactor flow, and thus attest the dynamic physiological performance of the proposed aortic valve model. To analyze the dynamic physiological performance, three scaffolds were tested at 60 BPM in a custom-made cardiac bioreactor designed to reproduce the physiological flow and pressure (aortic and ventricular) profiles (Figure 7). The normalized flow rate measured is presented in Figure 7a. It is the average flow rate for the three scaffolds with 12 cardiac cycles for each scaffold. Regurgitation and leakage were also observed during diastole. They are due to the large orifice area between the three leaflets (Figure 5b and Figure 6b). The overall shape of the measured flow profile was similar to the physiological flow rate profile, and the standard deviation between scaffolds in the ascending flow phase was small. Figure 7b presents the averaged normalized pressures. Note that the aortic valve scaffolds could not withstand the transvalvular pressure gradient imposed at closure. The presence of a transvalvular pressure gradient caused the inversion of the leaflets during closure and their deterioration. The slower deceleration phase could also be explained by the absence of a transvalvular gradient, the absence of mechanical heterogeneity of the aortic valve scaffolds material, and possibly its geometry. Nonetheless, the overall flow profile was similar to the physiological one, and there were little variations between the three aortic valve scaffolds, especially during the systolic phase.

This biofabrication method is intended for the engineering of aortic valves using in vitro culture methods when cells will be added into a hydrogel mixture, optimized for cell viability, extracellular matrix production, etc. We predict that the expected mechanical properties of the tissue would develop over time, relying on gradually increasing the dynamic stimulation provided by the cardiac bioreactor, which will stimulate cellular activity and the production of extracellular matrix. The alginate would then be degraded in a controlled manner under these physiological conditions [44] to be replaced by the extracellular matrix produced by the cells. Therefore, the presented aortic valve hydrogel scaffold is not intended to be directly implanted in a patient, but rather after the cell-populated hydrogel scaffold will demonstrate flow profiles and a durability similar to the native aortic valve after an extended cell culture period under dynamic conditions. Thus, the assessment of the mechanical properties of the scaffolds presented here was essential to validate the fabrication method and confirm that the aortic valve model morphology is adequate to reproduce the physiological functionality of the native aortic valve.

## 3. Materials and Methods

### 3.1. Aortic Valve Geometries

The aortic valve scaffold was modelled (Figure 5), using the computer-aided software Solidworks 2021, according to references devoted to the anatomy of human aortic valves (see Table 1). The goal was to create a valve model that can be easily adjusted to the aortic root dimensions of a patient waiting for a heart valve replacement following CT (computed tomography) or MR (magnetic resonance) imaging of its aortic root. A 26 mm base diameter was chosen has a reference. The geometry was parameterized with the parameters presented in Table 1. Parameters with a value outside the range of the native valve are due to modifications needed to allow either the printing of the valve mold, to improve the injection of the hydrogel solution into the mold, or to ensure the durability of the alginate-based hydrogel scaffold in the cardiac bioreactor. The three leaflets of the model are identical as opposed to the human anatomy [54].

### 3.2. Sugar Glass Mixture of the Molds

Sucrose (crystalline), dextrose (D-glucose, anhydrous), and calcium chloride (CaCl_2_, anhydrous) were purchased from Fisher Scientific (Waltham, MA, USA). Distilled water was used in all solutions.

The preparation of the sugar glass mixture was inspired by previous works [60,61]. At first, 78 g of sucrose were dissolved in 50 mL of pyrogen-free water, stirred on a stirring/heating plate (SCILOGEX MS7-H550-Pro) at 400 RPM, and heated at 165 °C. Meanwhile, 5 g of sucrose, 1.2 g of dextrose, and 0.3 g of CaCl_2_ were added to 8 mL of water and then stirred on a stirring/heating plate at 200 RPM to form a second mixture. Once the first mixture had reached 120 °C, the second mixture was heated at 140 °C. Once the first mixture had reached 165 °C, it was removed from the plate to cool down at room temperature. Once the second mixture had reached 130 °C, it was removed from the plate and mixed with the first mixture. The final mixture was then poured into the printer glass syringe of the 3D printer as described in [61].

### 3.3. 3D printing of the Aortic Valve Sugar Glass Mold

The 3D printer used to print the sugar glass molds is described in [61]. Briefly, the 3D printer X, Y, Z positioning system was based on a commercial 3D printer (AW3D XL), and the original printer head was replaced by a custom-designed printer head for additive manufacturing of sugar glass. An open-source software (Slic3r, version 1.3.0) was used to slice the STL (stereolithography format) of the aortic valve digital mold model and generate a specific G-code program. The parameters used to slice the model were: 0.2 mm layer height, 1.85 mm/s printing speed, concentric infill pattern, 0.1% infill density, 0.45 mm extrusion width, 1 mm filament width, 15% overlap, and no support. The generated G-code program allowed precise control of the X, Y, Z positions, feed and extrusion rates, temperatures, the sugar glass mixture and printer’s head nozzle, and air-cooling (0–5 Psi air pressure), making possible the printing of the sugar glass molds [60,61]. Repetier-Host, an open-source software (version 2.2.2), was then used to control the printer’s Arduino Mega2560 micro-controller that interprets and executes the G-code commands, using Marlin firmware. The temperature of the printing head containing the syringe with sugar mixture and the 0.5 mm extrusion nozzle was maintained at 92 °C throughout printing. The printed aortic valve sugar glass mold had an average thickness of 1.5 mm. The mold was printed on a custom designed injection module made of polycarbonate (Figure 1a). Figure 2b,c show the longitudinal section and the vertical inner section of the mold, respectively. Finally, the mold was placed inside a culture incubator (37 °C, 100% relative humidity) for 3 min and filled with a 30 mL syringe loaded with 70% ethanol solution using the injection module for 4 min prior to the casting of the alginate (Section 3.5). A total of nine aortic valve sugar glass molds were printed to fabricate the nine aortic valve hydrogel scaffolds analyzed. Three scaffolds were used for each evaluation: geometric evaluation (Section 3.6), mechanical testing (Section 3.7), and physiological assessment of the functionality (Section 3.8) of the aortic valve scaffold model. Molds were then stored at 4 °C into an airtight packaging with moisture absorbing silica gel for use within a week.

### 3.4. Sodium Alginate Scaffold Formulation

The sodium-alginate hydrogel was prepared from low-viscosity alginate (W201502, M/G ratio = 1.56, Sigma-Aldrich, St. Louis, MO, USA). D-glucono-δ-lactone (GDL) was obtained from Sigma-Aldrich, St. Louis, MO. Calcium carbonate nanoparticles (CaCO_3_, 15–40 nm, 97,5%) were from Skypring Nanomaterials, Houston, TX. Pyrogen-free water was used in all solutions. All solutions were kept at 37 °C prior to mixing.

A solution of 1.74% *w*/*v* alginate (12.9 mL) was prepared and mixed at room temperature for 24 h. To initialize gelation (internal gelation), 0.714 mL of 45 mM nano CaCO_3_ solution as a source of calcium ions and 1.43 mL of 90 mM GDL were used. A molar ratio of 0.5 was used to achieve a neutral pH value. The CaCO_3_-GDL system allows a time-delayed release of crosslinking calcium ions. This facilitates injection of the alginate hydrogel formulation into the complex mold geometry before gelation occurs [46,60]. The CaCO_3_ suspension was added to sodium alginate solution and vortexed for 15 s. The GDL solution was then added to the calcium-alginate solution and vortexed for 10 s. The concentration of alginate in the aortic valve scaffolds was 1.5% *w*/*v* and the volume of the valve was 11.7 mL. An additional 3.3 mL of alginate solution was needed to make up for losses associated with the manufacturing process of the scaffold.

### 3.5. Fabrication of the Aortic Valve Scaffolds

The fabrication of the aortic valve scaffolds is schematized in Figure 1d–f. The sugar glass mold is filled with the sodium alginate scaffold formulation using the injection module (Figure 1d). Once the gelation of the hydrogel is completed, the filled sugar glass mold was dissolved using a custom-made dissolution module (Figure 1e). The dissolution module was filled with a 50 mM CaCl_2_ aqueous solution. The dissolution module was connected to three 500 mL culture media bottles (one for each inlet/outlet combination) filled with the same 50 mM CaCl_2_ aqueous solution. A peristaltic pump (Ismatec BVP, Wertheim, Germany) then provided a 90 mL/min closed loop circulation between the dissolution module and the three 500 mL bottles containing the CaCl_2_ solution (not shown). The dissolution module was filled before the incorporation of the sugar mold/hydrogel with the 1 500 mL total volume of the CaCl_2_ aqueous solution.

### 3.6. Geometric Evaluation of the Aortic Valve Scaffold

Measurements of the 3D geometry of three aortic valve hydrogel scaffolds were obtained using micro-CT (TDM-X, eXplore Locus 80, GE Healthcare, Milwaukee, WI, USA) at 89 μm voxel size, 40 kVp, 100 mA, 0.5° angle increment, 90 ms exposure time, binning detector 4 × 4 and an average picture of 3. Resulting scans were reconstructed, segmented via intensity thresholds, and then exported into stereolithography (STL) geometries using MicroView (Parallax Innovations, version: 2.5.0, Ilderton, ON, Canada). The reconstructed scaffolds and the original model were manually overlapped. Slices were then made at the sino tubular junction, the mid-sinus, and the base for comparison with the original dimensions. The scaffolds were stored inside a 10 mM CaCl_2_ aqueous solution at 4 °C prior to the scans for less than 24 h to maintain their geometry [62], since the micro-CT apparatus to perform the evaluation was not immediately available after the fabrication of the scaffolds.

### 3.7. Mechanical Testing of the Aortic Valve Scaffolds

Uniaxial compression testing was performed to measure the elastic modulus of the sodium-alginate hydrogel scaffolds with an Instron E1000 apparatus using a 10 N capacity load cell. The accuracy was ±0.0025 N. The mechanical testing was performed at a crosshead speed of 1 mm × min^−1^ for a total compression of 30% of 8 mm in diameter samples taken under the base (Figure 3a). Three samples were tested for each of the three aortic valve hydrogel scaffolds dedicated to the mechanical testing.

### 3.8. Physiological Assessment of the Functionality of the Aortic Valve Scaffold

The flow and pressure (aortic and ventricular) profiles of three aortic valve scaffolds were obtained using a custom-made cardiac bioreactor specially designed to reproduce the exact environment of the heart. The bioreactor is described in [63,64]. Note that the scaffolds were stored in a 10 mM CaCl_2_ solution prior to the functional evaluation for less than 24 h at 4 °C.

## 4. Conclusions

This study established the feasibility of a method to manufacture sodium-alginate aortic valve scaffolds that possess a native-like geometry. The method uses sugar glass printed molds for the rapid casting of the scaffolds. It is a relatively simple and low-cost method that could help to make personalized tissue-engineered heart valves more accessible to patients in need of valve replacement. The reliability and repeatability of the scaffolds’ manufacturing still need to be further tested. The subsequent steps will be to optimize the integration of cells inside the hydrogel formulation, to develop protocols that will ensure cell viability, and to remodel the scaffold into a tissue-engineered heart valve following dynamic culture in the cardiac bioreactor.

## Figures and Tables

**Table 1:** Dimensions of the aortic valve scaffold.

Parameters	Value	Literature Range	Description
$R_{b}$	13 mm	11.3–14 mm	Radius of the base
$R_{c}$	0.9 $R_{b}$	0.78–1.0 $R_{b}$	Radius of the commissures
H	1.24 $R_{b}$	1.34–1.5 $R_{b}$	Valve height
$H_{s}$	0.5 $R_{b}$	0.71 $R_{b}$	Commissures height
$C_{c}$	0.26 $R_{b}$	0.20–0.26 $R_{b}$	Coaptation height
$d_{s}$	1.61 $R_{b}$	1.32–1.4 $R_{b}$	Radius of the outermost wall of the sinus
$h_{s}$	1.4 $R_{b}$	1.52–1.96 $R_{b}$	Sinus height
$h_{l}$	20 mm	−	Extension of the aortic valve wall
ϕ	25°	29–51°	Angle of the free edge to the plane through the three commissures
α	22°	15–25° [55] *	Angle of the bottom surface of the leaflet to the plane through the three commissures
$L_{f}$	1.92 $R_{b}$	2.42–2.48 $R_{b}$	Length of the leaflet free edge
$L_{h}$	1.41 $R_{b}$	1.2–1.4 $R_{b}$ [55,56] *	Length of the leaflet in the radial direction
$e_{l}$	1.5 mm	0.25–1.33 mm [57] **	Leaflets thickness
$e_{b}$	2.0 mm	0.6–1.977 mm	Thickness at the base
$e_{c}$	1.5 mm	1.824–2.138 mm	Thickness at commissures and sinus ends
$e_{o}$	1.5 mm	2.128–2.137 mm [58]	Thickness at the base of the ascending aorta
$R_{fo}$	2.9 mm	−	Length between the center of the aortic valve and the leaflet
θ	8.3°	− [59]	Angle between the commissure and the center of the aortic valve

* Dimensions correspond to an aortic valve subjected to a pressure of 100 mmHg. ** Thinnest to thickest section.

## Data Availability

All relevant data are within the manuscript.
